# Proposing a unified Mediterranean diet score to address the current conceptual and methodological challenges in examining adherence to the Mediterranean diet

**DOI:** 10.3389/fnut.2025.1533176

**Published:** 2025-09-12

**Authors:** Nahla Hwalla, Antonia Trichopoulou, Jacques Delarue, Felice Adinolfi, Furio Brighenti, Barbara Burlingame, Roberto Capone, Sandro Dernini, Maroun El Moujabber, Marcela González-Gross, Yari Vecchio, Nour Massouh, Farah Naja

**Affiliations:** ^1^Department of Nutrition and Food Sciences, Faculty of Agricultural and Food Sciences, American University of Beirut, Beirut, Lebanon; ^2^Academy of Athens, Athens, Greece; ^3^ER 7479 SPURBO, Faculty of Medicine, University of Brest, Brest, France; ^4^Department of Veterinary Medical Sciences, University of Bologna-Alma Mater, Bologna, Italy; ^5^Department of Food and Drug, University of Parma, Parma, Italy; ^6^Riddet Institute, Massey University, Palmerston North, New Zealand; ^7^International Center for Advanced Mediterranean Agronomic Studies (CIHEAM), Valenzano, Italy; ^8^ImFINE Research Group, Department of Health and Human Performance, Facultad de Ciencias de la Actividad Física y del Deporte-INEF, Universidad Politécnica de Madrid, Madrid, Spain; ^9^Department of Clinical Nutrition and Dietetics, College of Health Sciences, Research Institute of Medical and Health Sciences (RIMHS), University of Sharjah, Sharjah, United Arab Emirates; ^10^Faculty of Agricultural and Food Sciences, American University of Beirut, Beirut, Lebanon

**Keywords:** Mediterranean diet, unified Mediterranean diet score, diet adherence, lifestyle, cultural dietary practices

## Abstract

A plethora of studies has documented the benefits of the Mediterranean diet (MedDiet) for both human and environmental health. At the core of these investigations lies the assessment of adherence to it. In this manuscript, we aim to examine existing original scores used to assess adherence to the MedDiet and propose a framework for a unified score to address current challenges and complement the existing scores. A literature search was conducted to identify original MED scores, excluding those derived from earlier scores. A total of nineteen original scores were identified and examined. At the conceptual level, across existing scores, the following issues were identified: inconsistencies in food items, lack of holistic lifestyle approaches with focus on food-based components, limited cultural specificity, absence of sustainability evaluations, and regional focus solely on economically developed countries. At the methodological level, the majority of scores were based on cutoffs set by the population-specific distributions of dietary intake. Such cutoffs may be in discordance with the dietary recommendations of the different food groups considered. In addition, the definition of “adherence” is inconsistent across the scores, making the interpretation and comparability of the prevalence of adherence another methodological challenge. As a result, a framework for a Unified Mediterranean diet Score (UMEDS) is proposed. This framework consists of 10 food groups (whole grains, fruits, vegetables, dairy products, fish, legumes, olive oil, nuts and seeds, poultry, and red meat). These food groups are the common denominators of a traditional Mediterranean diet. In addition to the food-related components, the UMEDS also addresses physical activity, sleep, conviviality, and culture-specific food consumption (mainly composite dishes based on olive oil). For each of these items, evidence-based cut-offs were proposed. The total score for the UMEDS ranges from 0 to 22 with higher scores indicating a higher adherence (≤12 poor adherence, 13–17 moderate adherence, ≥18 good adherence). By integrating key components of dietary intake, lifestyle habits, and cultural practices, the UMEDS provides a comprehensive unified approach that aligns with global health guidelines and reflects the true spirit of the Mediterranean diet, rooted in food, lifestyle, culture, lifestyle, and traditional knowledge and practices.

## 1 Introduction

The Mediterranean diet (MedDiet) refers to the eating and lifestyle habits that were prevalent among populations living in countries located around the Mediterranean shores, started in the early 1960s (1), even though elements of the diet originated in antiquity (2). As defined and published by UNESCO, the MedDiet is derived from the Greek word “díaita,” which means lifestyle, and is recognized as a social practice that encompasses not only food consumption but a broader lifestyle in which regular physical activity plays an integral role (3). The traditional Mediterranean diet is characterized by a high intake of vegetables, legumes, fruits and nuts, and cereals (that in the past were largely unrefined), and a high intake of olive oil but a low intake of saturated lipids, a moderately high intake of fish (depending on the proximity of the sea), a low-to-moderate intake of dairy products (and then mostly in the form of cheese or yogurt), and a low intake of meat and low-to-moderate intake of poultry (depending on the access to the fish) (4). Interest in this diet was sparked by Prof. Keys from the University of Minnesota and Prof. Bergami from the University of Naples, who, back in the 1950's, observed the rarity of incidence of heart attacks in the population of Naples. Following these observations, the Seven Countries Study, consisting of 16 prospective cohorts in seven different countries (Greece, Italy, Japan, Finland, the former Yugoslavia, the Netherlands, and the US) was published. The Mediterranean diet was initially considered as a low saturated lipid diet conveying protection against coronary heart disease by lowering plasma cholesterol levels; however, current evidence indicates that its cardiovascular benefits are multifactorial, resulting from the combined effects of high intakes of unsaturated fats, fiber, polyphenols, and antioxidants (5–7). Since then, a vast body of scientific literature has documented several health benefits of the MedDiet, including a reduced risk of chronic health conditions such as cardiovascular disease (CVD), type 2 diabetes, neurodegenerative diseases, and some cancers, while promoting longevity and improving quality of life (8–12). More specifically, a more recent comprehensive review confirmed these findings by showing that adherence to MedDiet was strongly associated with reduced age-related chronic diseases (21.5%), neurological disorders (19%), and obesity-related metabolic features (12.65%), followed by CVDs (11.4%), cancer (10.1%), diabetes (7.5%), liver health (6.3%), inflammation (5%), mortality (5%), and renal health (1.2%) (13). Since the turn of the century and with the release of the sustainable development goals in 2015, a paradigm shift took place in the evaluation of dietary intake, whereby the focus extended beyond health to reach environmental and socioeconomic aspects of food consumption. In this context, the MedDiet has been extensively studies for its various sustainability dimensions and has consistently been shown to have a rich biodiversity, lower environmental impacts, high socio-cultural food value, and positive local economic returns (14, 15). The diet's emphasis on plant-based foods contributes to its sustainability, as it reduces greenhouse gas emissions and lowers the overall environmental footprint. Additionally, it promotes the consumption of local and seasonal produce and embraces sociocultural traditions making it a holistic approach to health and sustainability (16).

Despite proving to be a prototype of a dietary pattern that is healthy and sustainable, rates of adherence to the MedDiet continue to decline in many countries of the Mediterranean region (17). The erosion of this diet is mostly taking place among youth, whereby the MedDiet is increasingly being replaced by Western types of diets, characterized by high intake of refined grains, red meat, added sugar, and ultra-processed foods (18). In Greece, youth adherence is low, reflecting a shift toward Westernized eating patterns (19). In Italy, evidence shows a significant drop in adherence, particularly in southern regions (20). In Lebanon, a low adherence among adolescents has been observed (21). These trends in food consumption fueled regional and national efforts in many countries to develop evidence-based interventions aimed at halting this nutrition transition and promoting a greater adherence to the MedDiet (22, 23).

At the core of studying the MedDiet, its health benefits, sustainability, prevalence and promotion is the science of assessment of adherence to this diet. In 1995, Trichopoulou et al. (24) created the first and the most frequently used Mediterranean diet score, following which a myriad of dietary scores were proposed, all based on the frequency of pattern-consistent and pattern-inconsistent food consumption, as well as compliance with recommended intake. However, these scores varied in content (i.e., the selection of food groups) and methodology (i.e., the scoring framework used, such as median-based cutoffs or MedDiet recommendations) as some did not consistently include the same food groups, often excluded traditional Mediterranean diet foods such as olive oil (25–28), and rarely incorporated cultural and lifestyle components (29). For example, the MEDLIFE score is among the few that explicitly included physical activity as part of adherence to the MedDiet (30).

The differences among the existing scores partially led to inconsistent findings and challenged comparability across different studies. To this end, few studies reviewed these scores addressing either conceptual (inclusion of specific foods and/or lifestyle habits) or methodological (methods of calculation and validation) aspects of the scores used (31–34). A summary of the currently available scores is presented in Table 1, which provides an overview of the included food groups, their respective contributions, and the inclusion of lifestyle factors and traditional food items in each score.

**Table 1:** Conceptual description of existing Mediterranean diet scores.

**Score name**	**MDQI^a^**	**RevisedMDScale^b^**	**MDP score^c^**	**MD score^c^**	**aMED score^d^**	**MedDietScore^c^**	**Med-DQI^e^**	**MDS^c^**	**rMED index^f^**	**ITALIAN-MED^g^**	**MLDS^h^**	**PREDIMED-MEDAS^i^**	**mMDS^j^**	**MEDLIFE^k^**	**MDSS^l, 18^**	**MED-style^c^**	**SMDQ^m^**	**MEDI-LITE^n^**	**New MDSS°**
Year	2001	2003	2004	2004	2005	2006	2006	2009	2009	2011	2011	2012	2012	2014	2015	2015	2016	2017	2021
Setting	France	Greece	Spain	Spain	United States	Greece	France	Chile	Spain	Italy	Spain	Spain	Spain	Spain	Spain	Japan	Italy	Italy	United States
Cereals	✓(+)	✓(+)	✓^2^ (sum of bread, pasta, and rice)	✓(+)		✓(+)	✓(+)	✓	✓(+)	✓(+) (pasta)	✓(+)			✓^12^	✓(+)			✓(+)	
Whole grains					✓(+)	✓(+)		✓(+)					✓(+)	✓^12^		✓^19^	✓(+)		✓(+)
Refined cereals and pastries	✓(+) (white bread and white pasta and breakfast cereal)										✓(–) (pastries)	✓(–) (pastries)		✓^12^ (pastries)	✓		✓(–) (white bread and rice)		
Olive oil	✓(+)		✓(+)			✓(+)	✓(+)	✓(+)	✓(+)	✓(+)	✓(+)	✓(+)	✓(+)	✓(+)	✓(+)		✓(+)	✓	✓(+)
Ratio MUFA/SFA		✓(+)			✓(+)											✓(+)			
Fruits	✓(+)	✓(+)	✓(+)	✓(+)	✓(+)	✓(+)	✓(+)	✓(+)	✓(+)	✓(+)	✓(+)	✓(+)	✓(+)	✓^13^	✓(+)	✓(+)	✓(+)	✓(+)	✓(+)
Vegetables	✓(+)	✓(+) (except potatoes)	✓(+)	✓(+)	✓(+) (except potatoes)	✓(+)	✓(+)	✓(+) (except potatoes)	✓(+) (except potatoes)	✓(+)	✓(+)	✓(+)	✓(+)	✓(+)	✓(+)	✓(+)	✓(+)	✓(+)	✓(+)
Starchy vegetables						✓(+) (potatoes)				✓(–) (potatoes)				✓(–) (potatoes)	✓(–) (potatoes)				✓(+) (potatoes)
Legumes		✓(+)	✓(+)	✓(+)	✓(+)	✓(+)		✓(+)	✓(+)	✓(+)	✓(+)	✓(+)	✓(+)	✓(+)	✓(+)	✓(+)	✓(+)	✓(+)	✓(+)
Nuts and seeds			✓	✓(+)	✓(+)			✓(+)	✓(+)		✓(+)	✓(+)	✓(+)	✓^14^	✓(+)				✓(+)
Fish and seafood	✓(+)	✓(+)	✓(+)	✓(+)	✓(+)	✓(+)	✓(+)	✓(+)	✓(+)	✓(+)	✓(+)	✓(+)	✓(+)	✓(+)	✓(+)	✓(+)	✓(+)	✓(+)	✓(+)
Eggs														✓^15^	✓(+)	✓^20^			
Milk and dairy products		✓(–)		✓(–)		✓(–)		✓^6^	✓(–)		✓(–)		✓(–)	✓^16^	✓(+)	✓^21^		✓(–)	✓^24^
Sweets and SSBs								✓(–) (sugar)		✓(–) (SSBs)	✓(–)	✓(–)		✓(–)	✓(–)				✓(–)
Red meat and/or meat products	✓(–)	✓(–)	✓(–)	✓(–)	✓(–)	✓(–)	✓(–)	✓^7^	✓(–)	✓(–)	✓(–)	✓(–)	✓(–)	✓(–)	✓(–)	✓(–)	✓(–)	✓(–)	✓(–)
Poultry						✓(–)					✓(–)	✓(+)	✓(–)	✓^17^	✓(+)	✓^22^			✓(–)
Alcohol		✓^1^	✓(+)	✓^3^	✓^4^	✓^5^		✓(–) (wine)	✓^9^	✓^10^	✓^11^ (wine)	✓(+) (wine)	✓(+) (wine)	✓(wine)	✓(+)	✓^23^	✓(+) (wine)	✓	
Cholesterol	✓(–)						✓(–)												
SFA	✓(–)						✓(–)			✓(–) (butter)		✓(–) (butter/margarine)							✓(–) (other fats)
USFA								✓^8^											
Inclusion of lifestyle factors														✓					
Inclusion of traditional/indigenous food	✓						✓	✓		✓		✓		✓	✓				

(+) Food groups considered positive in the score.

(–) Food groups considered negative in the score.

^a^Mediterranean diet quality index.

^b^Revised Mediterranean diet scale.

^c^Mediterranean Diet score.

^d^Alternate Mediterranean diet index.

^e^Mediterranean diet quality index.

^f^Relative Mediterranean diet.

^g^Italian Mediterranean index.

^h^Mediterranean-like diet score.

^i^Precention with Mediterranean diet adherence score.

^j^Modified Mediterranean diet score.

^k^Mediterranean lifestyle index.

^l^Mediterranean diet serving score.

^m^Short Mediterranean diet questionnaire.

^n^Mediterranean diet score based on the literature.

°New Mediterranean diet scoring system.

^1^A value of one was assigned to men who consumed between 10 and 50 g per day and to women who consumed between 5 and 25 g per day (25).

^2^One point is added when either consumption of both white bread and rice is low or when consumption of whole-grain bread is high (41).

^3^Red wine consumption was computed as alcohol intake from red wine (0 and >20 g of alcohol = 1, and up to 20 g of alcohol = 3) (26).

^4^Assigned one point for alcohol intake between 5 and 25 g/day (27).

^5^Assigned score five for consumption of < 300 ml per day, score 0 for consumption of more than 700 ml per day or for no consumption, and scores four to one for consumption of 300–400, 400–500, 500–600, and 600–700 ml per day (100 ml = 12 g ethanol) (42).

^6^Assigned positive score for low fat and fermented dairy products and negative score for full fat dairy products not fermented (44).

^7^Assigned positive score for lean meat and negative score for fatty meat and processed meat (44).

^8^Positive for 4–8 teaspoons per day, negative for >8 or < 2 teaspoons per day, and half-point for 2–4 teaspoons per day for vegetable oils; positive for avocado (44).

^9^Two points were assigned for moderate consumers (5–25 g/day for women and 10–50 g/day for men) and 0 points for above and below the sex-specific range (45).

^10^One point for intake up to 12 g/day; abstainers and persons who consumed > 12 g/day received a 0 (46).

^11^Moderate red wine consumption (up to 20 g) was included as a favorable component while exceeding the upper limit or reporting no red wine consumption was coded as 0 (47).

^12^Positive for 3–6 servings/day: one serving = 40 g of white and whole grain bread, one serving = one plate for cereals), negative for pastries (30).

^13^Positive for three to six servings/day (30).

^14^Positive for one to two servings/day for nuts and olives (30).

^15^Positive for two to four servings/week (30).

^16^Positive for two servings/day for low-fat dairy products (30).

^17^Positive for two servings/week (30).

^18^Different positive scores were given for different food groups: three for fruits, vegetables, cereals, and olive oil, two for nuts and dairy products, and one for legumes, eggs, fish, white meat, and fermented beverage (wine and beer) (50).

^19^Positive for three to seven servings per day (28).

^20^Positive for two to three times per week (28).

^21^Positive for 1.5–2.5 servings per day (28).

^22^Positive for two to three times per week (28).

^23^Positive for 10–30 g per day for men and 5–15 g per day for women (28).

^24^Positive for 14–18 servings/week of low-fat and non-fat dairy and ≤ 6 servings/week for regular dairy (53).

In this manuscript, we aim to (1) describe available scores used in assessing adherence to the MedDiet, highlighting both the conceptual and methodological gaps and inconsistencies, and opportunities; and (2) propose a framework for a unified comprehensive score addressing the challenges, building on opportunities, and complementing existing scores.

## 2 Evaluation process

The development of the proposed unified MedDiet score was carried out through a structured process to ensure scientific rigor and applicability. This process included four steps. First, a systematic search and review of existing scores was performed. Second, the content and methodology of these scores were evaluated. Third, a series of collegial discussions among experts was held to refine and reach consensus on the essential components. Finally, the new score was formulated based on the evidence gathered and the experts' comments and discussions.

### 2.1 Review of previous scores

A systematic search across databases (PubMed, Scopus, Web of Science, Cochrane Library, Google Scholar) identified studies that developed methods for measuring adherence to the Mediterranean diet. A combination of Medical Subject Headings (MeSH) and free-text terms related to the Mediterranean diet and adherence scoring methods were applied for the search strategy. Key search terms included “Mediterranean diet,” “adherence,” “scoring,” “index,” “score,” “assessment,” and “measure.” Inclusion criteria focused on peer-reviewed studies with original scoring methods, while exclusion criteria filtered out non-English articles, reviews, and studies lacking clear scoring descriptions.

### 2.2 Comments on the various scores

A total of 19 Mediterranean diet scores were identified and reviewed. Each score was examined in relation to the food groups included, scoring units and cut-offs, the incorporation of lifestyle and cultural elements, and the scoring methodology used to assess adherence to the MedDiet. This review highlighted both conceptual and methodological opportunities and challenges, such as inconsistencies in food item inclusion, reliance on population-specific cut-offs (medians and tertiles), and limited integration of lifestyle factors.

### 2.3 Collegial discussions

The findings of the literature review were discussed within the Joint Task Force established by the International Center for Advanced Mediterranean Agronomic Studies (CIHEAM). The task force weighed the strengths and limitations of existing approaches and reached agreement on the guiding principles to be used to develop a new unified score.

### 2.4 Development of the new score

Following the review of existing scores, collegial discussions, the evidence and expert input were synthesized to formulate the Unified Mediterranean Diet Score (UMEDS). All identified scores were first examined, their strengths and limitations were debated among the experts, and comments were compiled. National dietary guidelines from Italy (35), Spain (36), Greece (37), and Lebanon (38) were also consulted to ensure that the framework reflected country-specific nutritional recommendations and cultural practices. However, to enhance comparability across populations, the final cut-offs used in the UMEDS were based primarily on international, evidence-based recommendations, namely the Mediterranean diet guidelines calculated by Germani et al. (39) and the EAT-Lancet Commission (15). Building on this appraisal, a draft framework was constructed and refined through rounds of discussion, to be finalized into a new score, the UMEDS, which is grounded in scientific evidence and expert consensus.

## 3 Scores used to assess adherence to the Mediterranean diet

The 19 identified scores were: MDQI (40), Revised MDScale (25), MDP score (41), MD score (26), aMED score (27), MedDietScore (42), Med-DQI (43), MDS (44), rMED index (45), ITALIAN-MED (46), MLDS (47), PREDIMED-MEDAS (48), mMDS (49), MEDLIFE (30), MDSS (50), MED-style (28), SMDQ (51), MEDI-LITE (52), and New MDSS (53). The review of these scores revealed both conceptual and methodological variability.

### 3.1 Conceptual challenges in existing Mediterranean diet scores

Existing Mediterranean diet scores have played an important role in assessing and quantifying adherence to the Mediterranean diet. These scores have allowed researchers to investigate the relationship between the Mediterranean diet and various health outcomes, contributing to a deeper understanding of its role in reducing the risk of noncommunicable diseases (NCDs). While these scores provide valuable insights into dietary patterns, they also present several conceptual challenges and opportunities.

#### 3.1.1 Inconsistency in food items across the scores

One major challenge was the inconsistency in food items across different Mediterranean diet scores which can affect the accuracy and comparability of adherence to the Mediterranean diet assessments. As detailed in Table 1, while scores consistently included core components such as fruits, vegetables, legumes, nuts and seeds, and fish, there was considerable variability in additional food items. For example, olive oil was featured in nearly all scores except four. Five scores included starchy vegetables (such as potatoes). Only eight of the 19 scores featured whole grains, 13 included cereals, and six addressed refined cereals and/or pastries. Variability also extended to the inclusion of fats and other dietary components: only five scores considered saturated fatty acids (SFA), while only one score included unsaturated fatty acids (USFA), and three accounted for the ratio of monounsaturated to saturated fatty acids (MUFA to SFA). Furthermore, poultry was included in eight scores, but only three considered eggs. Seven scores included sweets and/or sugar-sweetened beverages, and all but three scores included alcohol consumption, with only two scores that addressed cholesterol. While the 16 scores that included alcohol, particularly wine, considered moderate consumption as a positive factor, recent evidence challenges this concept. The World Health Organization (WHO) has stated that “no level of alcohol consumption is safe when it comes to human health” (54).

#### 3.1.2 Lack of a comprehensive lifestyle approach with focus on food-based components in the scores

Another limitation in many of the Mediterranean diet scores was their focus on food intake without incorporating a comprehensive lifestyle approach. This was a challenge because adherence to the Mediterranean diet encompasses more than just dietary choices; it is deeply rooted in the lifestyle practices of Mediterranean regions, including physical activity, stress management, and social interactions, which are integral to the Mediterranean lifestyle and significantly impact overall health (55, 56). Recent research underscored the significant relationship between nutrition, sleep quality, and overall health (29). With the exception of the Mediterranean Lifestyle Index (MEDLIFE) score, which incorporated lifestyle factors, such as physical activity, social habits, and conviviality (30), all other Mediterranean diet scores, however, focused solely on food intake.

#### 3.1.3 Limited cultural specificity in Mediterranean diet scores

A notable gap in current Mediterranean diet scores was their lack of cultural specificity, which limited the effectiveness of assessing dietary habits within the cultural context of different populations (29). Of note that the Greek Med Diet Score of 2003 (Revised MDScale), was assigned to each of nine indicated components with the use of study and gender-specific median values as the cutoff (25). Although several Med-type scoring systems had made efforts to incorporate traditional food items, such the MEDLIFE score, based on the Spanish Mediterranean food guide, which included eggs (30), these scoring systems primarily focused on food items rather than indigenous cooking recipes. For example, the Prevention with Mediterranean Diet Adherence Score (MEDAS) featured pasta, rice, and dishes seasoned with sofrito, a sauce made with tomato, onion, leek, or garlic, cooked with olive oil (48). The Mediterranean Diet Serving Score (MDSS), another Med-type score, validated within a female population from southern Spain, also accounted for potatoes (50). The Italian Mediterranean score (ITALIAN-MED) included potatoes as well (46). The Mediterranean-type Diet Score (MDS) diet score, developed in Chile, integrated avocadoes, a principal component of the Chilean diet (44). French diet scores, like the MDQI and Med-DQI (Mediterranean Diet Quality Index), factored in cholesterol and saturated fats (40, 43). However, as Godos et al. (29) argue, an underrated aspect of the Mediterranean diet relates to the consumption of foods using indigenous cooking methods and recipes. Without considering traditional culinary practices, dietary assessments may overlook the social and cultural elements that define the Mediterranean diet (57).

#### 3.1.4 Mediterranean diet scores originated from a regional focus by economically developed countries

Another significant gap in the existing Mediterranean diet scores was their geographic and socio-economic bias, as most originated from the economically developed countries around the Mediterranean Basin. Specifically, as shown in Table 1, 15 of the 19 scores originated from Spain, Italy, France or Greece, while the remaining Med-type scores originated from countries like Chile, the United States, China, and Japan. This limited scope overlooked the fact that the Mediterranean diet, by definition, is rooted in the traditional eating habits of all countries bordering the Mediterranean Sea, including both develop and developing nations of the southern Mediterranean basin (58, 59). As such, the available scores did not fully capture the diversity of dietary patterns across the different countries of the Mediterranean basin which belong to varying levels of socio-economic and cultural contexts. It is noteworthy that the original Mediterranean diet pyramid specifically includes whole grains, such as bread, pasta, rice, couscous, polenta and bulgur (1). The predominance of scores originating from developed countries can lead to a less comparable assessment of adherence to the Mediterranean diet, as countries outside this developed group may have specific dietary habits and cultural practices that were not adequately represented by these existing scores (60).

### 3.2 Conceptual opportunities in existing Mediterranean diet scores

Another valuable opportunity in the existing Mediterranean diet scores was the consistency in the inclusion of many common foods. Despite the variability in a number of food groups, there were several core components that frequently appeared across different scores, such as olive oil, fruits, vegetables, legumes, nuts and seeds, and fish. This commonality reflected a shared understanding of the fundamental elements of the Mediterranean diets and highlighted the diet's score strengths (1, 4, 5). The widespread inclusion of these foods indicated a consensus on their importance for health and wellbeing, reinforcing the diet's validity as a model for nutrition (12–14, 61).

### 3.3 Methodological challenges in Mediterranean diet scores

Table 2 provides a comprehensive overview of the different methodologies used in Mediterranean diet scores, highlighting the associated challenges and opportunities.

**Table 2:** Methodological description of existing Mediterranean diet scores.

**Score**	**Cutoff**			**Unit**	
	**Median**	**Consumption (tertiles, quartiles, percentiles, etc.)**	**MD recommendation**	**Grams**	**Servings**
MDQI		✓		✓	
Revised MD scale	✓			✓	
MDP score		✓			✓
MD score		✓		✓	
aMED score	✓			✓	
MedDietScore			✓		✓
Med-DQI		✓		✓	
MDS			✓		✓
rMED index		✓		✓	
ITALIAN-MED		✓		✓	
MLDS		✓		✓	
PREDIMED			✓		✓
mMDS		✓			✓
MEDLIFE			✓		✓
MDSS			✓		✓
MED-style			✓		✓
SMDQ			✓		✓
MEDI-LITE		✓		✓	
New MDSS			✓		✓

#### 3.3.1 Different scoring frameworks used in calculating the Mediterranean diet scores

The calculation of Mediterranean diet scores across the various scores presented its own set of challenges, primarily due to the use of different units of measurement and statistical approaches. For example, scoring frameworks such as those for the MDQI and rMED (relative Mediterranean Diet) index measured food intake in grams, while others like the MEDAS and MedDietScore (Mediterranean Diet Score) used servings (Table 2). Moreover, the statistical methods employed—ranging from median calculations, tertiles, quartiles, and percentiles of food consumption to Mediterranean dietary guidelines—further contributed to variability. Scores using median-based methods, such as the revised MD scale and the aMED (alternate Mediterranean Diet Index) scores, were less sensitive to dietary variations compared to those using tertiles or percentiles—like the MDQI, Med-DQI, and MEDI-LITE (Mediterranean Diet Score based on the literature)–which provided more nuanced insights but introduced variability based on population stratification. The use of median and tertiles-based scoring methods can be particularly problematic in developing countries, where median consumption levels for Mediterranean diet foods may fall below recommended amounts (28, 62). As a result, these scores risked overestimating adherence if the overall intake of the population was inherently low (34, 60). On the other hand, scores such as the MedDietScore, MDS, MEDAS, MEDLIFE, MDSS, MED-style, SMDQ (Short Mediterranean Diet Questionnaire), and new MDSS, which were based on the principles of Mediterranean diet recommendations (Table 2), offered a more objective and consistent scoring framework for assessing adherence, addressing some of the limitations of other scores (48, 59).

#### 3.3.2 Complexities in interpreting adherence to the Mediterranean diet

A significant challenge in the available scores was the variability in the definition of what constituted “good” adherence. For instance, the MEDAS employed a scoring system where participants who achieved scores of 8–9 or ≥10 out of a possible 14 points (57%−64% or ≥71%) were classified as having high adherence to the Mediterranean diet, while those scoring ≤ 7 (≤ 50%) were categorized as having low adherence (48). In contrast, the MDSS designated individuals with a score of 16 or higher, out of a possible 24 points (67% or above), as adherent, applicable to both adults and elderly populations (50). This disparity in scoring presented a challenge in comparing results across different studies, as the same level of adherence may be classified differently. For example, an individual who scored 60% of the points would be considered highly adherent according to MEDAS but not adherent according to the MDSS. Such variability complicated cross-study comparisons and made it difficult to establish consistent conclusions about adherence to the Mediterranean diet.

### 3.4 Methodological opportunities in existing Mediterranean diet scores

#### 3.4.1 Leveraging Mediterranean diet scores for health outcomes improvement

The availability of diverse Mediterranean diet scores presented a significant opportunity due to their role in helping achieve a correlation between specific dietary intakes with various health outcomes. Many of these scoring systems have been shown to correlate with positive health indicators, such as reduced risk of cardiovascular diseases, improved metabolic profiles, and better overall health status (25, 27, 28, 45, 46, 51). For example, adherence to the Mediterranean diet as measured by the rMED index was associated with a significantly reduced risk of coronary heart disease (45). Lower adherence to the Mediterranean diet as measured by the SMDQ was associated with gastrointestinal symptoms (51). By utilizing these scores, researchers and healthcare professionals were able to effectively monitor dietary patterns and provide public health recommendations that align with the Mediterranean diet's proven health advantages.

#### 3.4.2 Ranking adherence to the Mediterranean diet

The diverse Mediterranean diet scores offered the valuable opportunity to rank individuals based on their adherence to dietary guidelines, facilitating a more nuanced understanding of dietary patterns within populations (31, 33). By categorizing individuals according to their scores, researchers and healthcare professionals were able to identify varying levels of adherence, from high to low, which was crucial for tailoring personalized interventions and monitoring progress (31, 60). This ranking capability enabled the detection of dietary trends and disparities, helping to pinpoint those who may benefit most from dietary modifications or support (18, 34). Furthermore, it allowed for the stratification of study populations in research settings, enhancing the ability to examine the relationships between levels of adherence and health outcomes (5, 25).

## 4 Proposed framework for a unified Mediterranean diet score

To address the challenges of previous scoring systems, the Unified Mediterranean Diet Score (UMEDS) is introduced as a comprehensive framework that integrates dietary intake, lifestyle habits, and cultural practices. This approach enhances the accuracy, cultural relevance, and consistency of adherence assessments, making it applicable across diverse populations.

The development of the UMEDS was conducted within the context of a broader initiative led by the Joint Task Force of the International Center for Advanced Mediterranean Agronomic Studies (CIHEAM), the Federation of European Nutrition Societies (FENS), and the International Union of Nutritional Sciences (IUNS), with contributions from Dr. Farah Naja and Nour Massouh. The three critical components of the proposed framework—dietary intake, lifestyle habits, and cultural practices—ensure that the Mediterranean diet's rich cultural diversity is respected and incorporated into the evaluation process. Table 3 provides a description of the scoring system proposed for the UMEDS, including the number of points corresponding to (1) intake of various food items, (2) lifestyle components, and (3) cultural factors, as will be detailed in this section.

**Table 3:** Description of the proposed framework for a unified Mediterranean diet score (UMEDS).

**Components**	**Scoring**	**Reference for recommendation**
**Food intake**		
Whole grains	0 = < 115 g	Germani et al. (230 g/day) (39)
	1 = 115 through 229 g	
	2 = ≥230 g	
Fruits	0 = < 225 g	Germani et al. (450 g/day) (39)
	1 = 225 through 449 g	
	2 = ≥450 g	
Vegetables	0 = < 200 g	Germani et al. (400 g/day) (39)
	1 = 200 through 399 g	
	2 = ≥400 g	
Dairy products	0 = < 140 g	Germani et al. (280 g/day) (39)
	1 = 140 through 279 g	
	2 = ≥280 g	
Fish	0 = >100 g	Eat-Lancet (0–100 g/day) (15)
	1 = ≤ 100 g	
Legumes	0 = < 50 g	Germani et al. (100 g/day) (39)
	1 = 50 through 99 g	
	2 = ≥100 g	
Olive oil	0 = < 15 g	Germani et al. (30 g/day) (39)
	1 = 15 through 29 g	
	2 = ≥30 g	
Nuts and seeds	0 = < 3.25 g	Germani et al. (6.5 g/day) (39)
	1 = 3.25 through 6.4 g	
	2 = ≥6.5 g	
Poultry	0 = >58 g	Eat-Lancet (0–58 g/day) (15)
	1 = ≤ 58 g	
Red meat	0 = >14 g	Germani et al. (14 g/day) (39)
	1 = ≤ 14 g	Eat-Lancet (0–14 g/day) (15)
**Lifestyle**		
Physical activity	0 = inactive (< 150 min/week)	WHO guidelines on physical activity and sedentary behavior (65)
	1 = moderately to highly active (≥150 min/week)	
Sleep	0 = < 7 or >9 h/night	Healthy living guide 2020/2021 (7–9 h per night) (66)
	1 = 7–9 h/night	
Conviviality (eating with family and friends)	0 = rarely/never (< 5 times per week)	de la Torre-Moral et al. (≥5 meals per week) (70)
	1 = regularly/frequently (≥5 times per week)	
**Culture**		
Intake of traditional/indigenous salad	0 = rarely/never (< 2 times per week)	
Total interpretation	22	Bloom's criteria (72, 73)
	18–22 = good adherence	
	13–17 = moderate adherence	
	≤ 12 = poor adherence	

### 4.1 Food intake evaluation

The UMEDS evaluates the intake of 10 key food groups: whole grains, fruits, vegetables, dairy products, fish, legumes, olive oil, nuts and seeds, poultry, and red meat. The intake cutoffs for each food group are based on the Mediterranean diet guidelines as calculated by Germani et al. (39) and Eat-Lancet recommendations (15). While many existing Mediterranean diet scores use portion-based measures, the UMEDS specifies intake in grams to ensure precision and comparability across populations, as portion sizes vary widely between cultures (63). This score is primarily designed to be used in epidemiological research, where visual aids, household measures, and increasingly accessible digital tools can help participants estimate their intake.

For whole grains, fruits, vegetables, dairy products, legumes, olive oil, and nuts and seeds, the UMEDS assigns 0–2 points depending on consumption levels: 0 points for intake below 50% of the recommended amount, one point for intake between 50 and 100%, and two points for meeting or exceeding 100%. For example, the recommended daily intake of whole grains is 230 g (39), with 0 points awarded for consumption below 115 g, one point for intake between 115 to 230 g, and two points for intake at or above 230 g.

For food groups with a maximum recommended intake—such as red meat, poultry, and fish-−0 points are given for consumption above the recommended limit, while one point is assigned for intake within the recommended range. For example, the maximum recommended daily intake for red meat is 14 g (15, 39), with 0 points awarded for consumption over 14 g and one point for intake between 0 and 14 g. These recommendations are in line with the Eat-Lancet Commission's recommendation (15) and the Mediterranean food pyramid of 14 g of red meat per day (39).

Acknowledging concerns regarding the adequate intake of bioavailable irons, particularly in the context of prevention and control of anemia among women of reproductive age, as emphasized by the WHO (64), the UMEDS incorporates several sources of bioavailable iron, including fish (100 g), poultry (58 g), legumes (100 g), whole grains (230 g), and nuts (6.5 g). Furthermore, the UMEDS also recognized that red meat intake can vary based on regional dietary patterns and traditional food practices, which are further detailed in the cultural component of the framework (Section 3, Paragraph 3).

By focusing on these common food groups, researchers and practitioners can ensure that key health-promoting elements are recognized and emphasized. The UMEDS prioritizes adherence to the Mediterranean diet with a focus on promoting health and excludes alcohol as a component, in line with the WHO's position that no amount of alcohol is considered safe for human health (54). By integrating key food groups universally acknowledged in Mediterranean diet literature, the score not only assesses adherence but also supports public health goals by encouraging dietary patterns associated with reduced risk of chronic diseases. This evidence-based alignment ensures that the UMEDS remains a powerful tool for promoting health outcomes through dietary interventions.

### 4.2 Lifestyle components

The UMEDS recognizes that adherence to the Mediterranean diet involves more than just food choices; it also encompasses key lifestyle habits that are integral to the Mediterranean way of life. This inclusion of lifestyle factors addresses a significant gap in previous Mediterranean diet scores, which often focused exclusively on dietary intake. By incorporating these lifestyle components, the UMEDS provides a more holistic assessment of adherence, aligning with recent research emphasizing the importance of integrating diet with other health-promoting behaviors. This approach enhances the score's ability to capture the full spectrum of the Mediterranean lifestyle, offering a more comprehensive evaluation of adherence.

Based on evidence-based recommendations from the World Health Organization and the Harvard T.H. Chan School of Public Health (65, 66), the UMEDS evaluates lifestyle habits by awarding points for two main lifestyle behaviors: physical activity and sleep. First, it awards one point for engaging in at least 150 min of exercise per week, reflecting the importance of regular physical activity as a cornerstone of the Mediterranean lifestyle and essential for overall health. While this cutoff is based on international and U.S. guidelines, we acknowledge that Mediterranean-specific reference criteria for physical activity are lacking. Developing Mediterranean-specific guidelines should be a priority for future research. Second, it allocates one point for obtaining between 7 and 9 h of sleep per night, emphasizing the significance of adequate rest as a vital component of wellbeing. Recent research underscores the significant relationship between nutrition, sleep quality, and overall health (29). In particular, evidence from diverse populations support a positive association between adherence to the Mediterranean diet and improved sleep outcomes, including longer sleep duration, better sleep quality, and fewer sleep-related problems (67–69).

Additionally, the UMEDS recognizes the importance of social interactions in the Mediterranean lifestyle. Conviviality, or the regular practice of eating with family and friends, is a hallmark of Mediterranean cultures and is closely associated with health benefits. The UMEDS awards 1 point for regularly eating with family and friends (five or more times per week). The threshold of five times per week was chosen based on research that identifies “optimal family meal frequency” as five or more meals per week, which is linked to improved health outcomes (70). This social component acknowledges that the Mediterranean diet is not only what is eaten but also about how and with whom meals are shared. Incorporating lifestyle factors into Mediterranean diet assessments is crucial for a more comprehensive understanding of adherence and its health implications. This comprehensive approach can lead to more accurate evaluations and more effective dietary recommendations.

### 4.3 Cultural component

Cultural practices play an important role in the Mediterranean diet, and the UMEDS addresses the need for cultural specificity in dietary assessments. Traditional and indigenous foods are not only a reflection of local culinary traditions but also an integral part of the Mediterranean diet. By integrating these cultural components, the UMEDS ensures that adherence evaluations respect and preserve the diverse culinary traditions within the Mediterranean diet framework. This cultural specificity therefore enhances the accuracy of adherence assessments while also promoting the diversity of Mediterranean dietary practices (71).

The UMEDS evaluates cultural adherence by awarding points for the regular consumption of traditional Mediterranean foods. When used, each country has the flexibility to indicate two items (a salad and a vegetable or legume dish cooked with olive oil) as indigenous foods. Such items ought to be consumed at least twice weekly. Accordingly, one point is given for consuming traditional salads, such as Greek salad in Greece or tabbouleh in Lebanon, at least twice per week. This emphasizes the importance of incorporating culturally specific dishes rich in vegetables and healthy lipids. Similarly, one point is given for consuming traditional Mediterranean main dishes, such as couscous in North African countries or pasta in Italy, at least twice per week. These main dishes typically include a combination of legumes, vegetables, fish, and other key components of the Mediterranean diet, reflecting the diverse and balanced nature of traditional Mediterranean cuisine across different countries.

By adopting a comprehensive and country-specific approach to dietary assessments, the UMEDS can help researchers gain a better understanding of the challenges and opportunities in promoting healthier eating patterns, especially in culturally diverse settings such as the Mediterranean region. Developing a more inclusive range of scores that represent both economically developed and developing Mediterranean countries is essential for capturing the full spectrum of Mediterranean dietary practices. This broader representation can enhance the accuracy of adherence assessments and provide more relevant insights into dietary patterns and health outcomes. By incorporating these traditional foods, the UMEDS enhances the cultural relevance of the score, ensuring that it captures the full range of Mediterranean dietary practices. This approach addresses the dominance of scores from economically developed countries, which has been a common problematic issue in previous Mediterranean diet assessments that primarily focus on dietary patterns from nations like Spain, Italy, and Greece. The UMEDS broadens the scope to include a wider range of Mediterranean food culture, making it a more inclusive and representative measure of adherence.

### 4.4 Scoring system and interpretation

The scoring system of the UMEDS is designed to provide a clear, consistent, and comparable measure of adherence to the Mediterranean diet, ensuring that comparisons and conclusions drawn from the score are both valid and meaningful. The total score can reach a maximum of 22 points, with adherence levels categorized based on Bloom's criteria (72, 73). A score between 18 and 22 points (equivalent to 80%−100%) signifies good adherence to the Mediterranean diet. Scores in the range of 13–17 points (60%−79%) reflect moderate adherence, while scores of 12 points or less indicate poor adherence.

A detailed interpretation of the scores reveals that high adherence, defined as 80% and above, indicates a good alignment with the Mediterranean diet. Individuals in this category consistently consume key foods, follow lifestyle habits, and engage in cultural practices that reflect the principles of the diets. Moderate adherence, ranging from 60 to 79%, suggests partial compliance with the Mediterranean diet. While participants in this range align with some aspects of the diet, there are notable areas where improvement is needed, highlighting the potential for dietary and lifestyle adjustments. Finally, poor adherence, categorized as below 60%, indicates that participants are not closely following the Mediterranean diet. This group may benefit from targeted interventions and educational efforts aimed at improving diet quality, lifestyle habits, and cultural practices related to the Mediterranean diet.

This structured ranking system allows for a nuanced evaluation of dietary patterns within populations, enabling researchers and healthcare professionals to identify individuals who closely adhere to the Mediterranean diet and those who may need additional support. By using clear cut-off points, the UMEDS enhances the ability to detect dietary trends and disparities, making it a valuable tool for both research and public health initiatives.

In addition to improving interpretability, the standardized scoring system simplifies the calculation of Mediterranean diet scores. By focusing on well-defined food groups, lifestyle factors, and cultural practices, with clear scoring thresholds, the UMEDS minimizes methodological variability and enhances the reliability of dietary assessments. This consistency ensures that the score can be applied across different populations, facilitating more reliable comparisons of adherence levels.

While the UMEDS aligns with evidence linking adherence to the Mediterranean diet to positive health outcomes, further validation is necessary to establish its direct association with reduced cardiovascular risk, diabetes, and other chronic diseases.

## 5 Strengths and limitations

The UMEDS offers several strengths. It represents the first framework to integrate dietary, lifestyle, and cultural components of the Mediterranean diet within a single score. By incorporating food groups common to existing scores while also adding lifestyle practices such as physical activity and culture-specific traditional dishes, the UMEDS seeks to reflect the UNESCO definition of the Mediterranean diet. Furthermore, its standardized scoring system, which is based on evidence-based cut-offs, may enhance comparability of adherence to the MedDiet across populations, improve consistency of results, and reduce methodological variability. In parallel, the UMEDS also has limitations that would be ironed out with future testing and validation. The score's point distribution and cut-off values are grounded in existing literature; however, they have not yet undergone validation. Another specific limitation relates to the use of grams rather than portion sizes for quantifying food intake. While the rationale for this choice was to enhance precision and comparability across populations, it may nonetheless reduce ease of application in practice, where portion-based guidance is often more feasible. However, the UMEDS is proposed as a framework for epidemiological research and is not intended for direct use in clinical practice. Additionally, we have noted that cut-offs for physical activity and sleep were based on international guidelines (WHO and Harvard) in the absence of Mediterranean-specific references, this remains a limitation as it may not fully account for cultural variability across Mediterranean countries.

## 6 Work in progress for the future

Future research is planned to explore the expansion of the UMEDS framework to incorporate broader sustainability aspects, using a multi-dimensional approach. This expanded framework could include ecosystem stability (e.g., greenhouse gas emissions, water and energy use) to address the environmental footprint of dietary choices, alongside food affordability and availability, and resilience metrics to evaluate food security under changing socioeconomic and climatic conditions, such as climate change, biodiversity loss, sustainable fisheries, food losses and waste (74, 75). Integrating these additional dimensions into the UMEDS would enhance its applicability to sustainable diet assessments.

## 7 Conclusion

In this paper, we evaluated existing Mediterranean diet scores for adults and proposed a unified framework that addresses their challenges, builds on their opportunities, and complements previous efforts. While available scores provided valuable insights into dietary patterns, they also presented several conceptual and methodological limitations. The key challenges identified included inconsistency in food item selection, a lack of lifestyle integration, limited cultural specificity, regional biases, and inconsistencies in the calculation methods and interpretation of what constitutes “good” adherence. The opportunities, on the other hand, were the alignment with nutrition guidelines for health promotion, the consistent inclusion of core food items of the Mediterranean diet, correlations with health outcomes, and the ability to rank individuals based on their level of adherence. The introduction of the UMEDS addresses the identified gaps and capitalizes on the opportunities by offering a more comprehensive, standardized, and culturally inclusive framework. By integrating key components of dietary intake, lifestyle habits, and cultural practices, the UMEDS could provide a comprehensive approach that not only aligns with global health and sustainability guidelines and goals, but also reflects the spirit of the Mediterranean diet, rooted in food, lifestyle, culture, and traditional knowledge and practices.
